# Investigating Stress and Coping Behaviors in African Green Monkeys (*Chlorocebus aethiops sabaeus*) Through Machine Learning and Multivariate Generalized Linear Mixed Models

**DOI:** 10.3390/vetsci12030209

**Published:** 2025-03-01

**Authors:** Brittany Roman, Christa Gallagher, Amy Beierschmitt, Sarah Hooper

**Affiliations:** 1 Center for Conservation Medicine and Ecosystem Health, Ross University School of Veterinary Medicine, Basseterre KN 0101, Saint Kitts and Nevis; 2 Department of Biomedical Sciences, Ross University School of Veterinary Medicine, Basseterre KN 0101, Saint Kitts and Nevis; 3 Department of Clinical Sciences, Ross University School of Veterinary Medicine, Basseterre KN 0101, Saint Kitts and Nevis; 4 Behavioural Science Foundation, Basseterre KN 0101, Saint Kitts and Nevis

**Keywords:** animal welfare, 3Rs, stress response, behavior, African green monkey, refinement, cortisol, lysozyme, β-endorphin, machine learning, multivariate generalized linear mixed models

## Abstract

Integrating behavioral and physiological assessment is critical to improve our ability to assess animal welfare in biomedical settings. Hair, blood, and saliva samples were collected from 40 recently acquired male African green monkeys (AGMs) to analyze concentrations of hair cortisol, plasma β-endorphin, and lysozyme alongside focal behavioral observations. The statistical methodology utilized machine learning and multivariate generalized linear mixed models to find associations between behaviors and fluctuations of cortisol, lysozyme, and β-endorphin concentrations. The study population was divided into two groups to assess the effectiveness of an enrichment intervention, though the hair cortisol results revealed no difference between the groups. The principal component analysis (PCA) with a Bayesian mixed model analysis reveals several significant patterns in specific behaviors and physiological responses, highlighting the need for further research to deepen our understanding of how behaviors correlate with animal welfare. This study’s methodology demonstrates a more refined approach to interpreting these behaviors that can help improve animal welfare and inform the development of better management practices.

## Simple Summary

Understanding both behavior and physical health is important for measuring how well animals are coping in captivity. In this study, we collected hair, blood, and saliva samples from 40 male African green monkeys (AGMs) (*Chlorocebus aethiops sabaeus*) to measure their stress responses. We used principal component analysis (PCA) with a Bayesian mixed model analysis to find patterns in their behaviors related to cortisol, lysozyme, and β-endorphin. While the animals were divided into two groups to see if an enrichment activity would help their welfare, there was no difference in their hair cortisol levels. The statistical analysis, however, shows certain behaviors were connected to stress, suggesting that we need more research to understand how factors like environmental and social interactions are connected to animal welfare. This study shows that looking closely at animal behaviors with advanced statistical techniques can provide better objective assessments of behavior, which can lead to better veterinary management practices.

## 1. Introduction

A research animal should be able to cope with the conditions in which it lives, therefore demonstrating a good state of welfare where it is healthy, comfortable, well-nourished, safe, able to express innate behavior, and is not suffering unduly [1]. Appropriate husbandry practices can greatly influence animal welfare in a variety of ways, including a strategy widely known as enrichment [1,2]. Enrichment is defined as “an animal husbandry principle that seeks to enhance the quality of captive animal care by identifying and providing the environmental stimuli necessary for optimal psychological and physiological well-being” [3]. Enrichment frameworks should be developed with knowledge of a species’ natural history, in situ activity budgets, and encouragement of species-specific behaviors [4,5,6,7]. Generally, the evaluation of animal welfare and enrichment effectiveness can be very difficult because of the influence of many different intrinsic and extrinsic variables, which are not all controllable, and the difficulty of measuring success [8].

There is considerable variability in the effectiveness of enrichment between non-human primate (NHP) species, and even within-species enrichment effectiveness can vary depending on factors such as age, sex, and temperament [2,6,9]. For example, rhesus females will engage with toys more than males, whereas long-tailed macaque males will engage with toys more than their female counterparts [10]. Many enrichment studies focus solely on whether animals engage with enrichment and equate usage to success [11]; however, this might not correlate with an actual reduction in stress [12]. Stress itself can be classified into categories based on the duration, source, and response [13]. This study focuses on chronic stress, which occurs when an animal is under prolonged exposure to stressors. Acute stress is more typically defined as a short-term reaction to an immediate stressor, one which alters physiological parameters, such as heart rate when the body activates the fight-or-flight response during the brief restraint of an animal [13]. Given these distinctions, evaluating enrichment should involve an integrated approach that examines both behavioral and physiological markers of stress. Since the early 2000s, research has expanded to examine enrichment’s impact on physiological parameters, principally glucocorticoids, such as cortisol, and continues to be a key area of study. [14]. The field of enrichment research is being challenged to strive for improved evaluation of animal welfare with holistic approaches that do not rely solely on the appearance or absence of a single behavior [5,6,7,12].

The accurate analysis of behavior as a whole is inherently complex. Successful veterinary management of NHP populations should utilize effective methods of behavioral assessment that mitigate anthropogenic biases and subjective interpretations [15]. Activity budgets are a common tool used to assess animal welfare by compartmentalizing different behaviors into categories. Activity budgets of animals in captivity are often compared to their wild counterparts with the assumption that similarity equates to better animal welfare; however, this has been challenged in a growing body of literature [16,17,18]. Certain forms of enrichment have been found to greatly benefit animal welfare that would not be found in nature, such as computer-based enrichment with NHPs [19] or co-housing animals with different species. In the wild, domestic cats and AGMs do not coexist but one pilot study showed positive results when housing AGMs with domestic cats [20]. If wildness is not the best assessment of welfare, then methodologies should evolve to identify better standards. An objective assessment can integrate the analysis of physiological parameters into behavioral assessments, and by leveraging tools like PCA analysis and multivariate models, associations between behaviors and physiological biomarkers can be observed.

Extensive literature documents cortisol’s utility as a stress biomarker as the end-product of the hypothalamic–pituitary–adrenal (HPA) axis [21]. Cortisol measurements provide an objective way to quantify stress in a way that may not be immediately apparent through behavioral observation alone. The use of cortisol as a welfare indicator has some limitations; therefore, it is important to recognize that other physiological markers may also be able to contribute to assessing an animal’s overall well-being [22,23,24,25,26]. Lysozyme, a microbial enzyme and key component of the innate immune system [27], was chosen due to its sensitivity to stress-induced immunosuppression. Research has demonstrated a negative correlation between salivary lysozyme levels and stress, likely due to the immunosuppressive effects associated with the stress response [28]. Chronic stress, in particular, has been linked to sustained reductions in immune function, suggesting lysozyme is a potentially valuable biomarker for assessing long-term welfare effects.

β-endorphin was also selected as a stress biomarker due to its role in modulating the HPA axis. Released simultaneously with adrenocorticotropic hormone (ACTH), β-endorphin helps attenuate the activation of the HPA axis. [29] and reduce pain perception, effectively raising an organism’s pain threshold [30]. In addition to these analgesic effects, β-endorphin has significant effects on stress-related responses and coping behaviors. In chronically stressed humans, cortisol is elevated, but β-endorphin levels are not, suggesting that β-endorphin has a bigger role in attenuating acute stress responses than chronic stress [30,31]. Additionally, β-endorphins are used to investigate the mental state of animals as a potential biomarker for mood disorders like depression [29,32,33,34,35]. β-endorphin and lysozyme are not directly related but both can be influenced by the same physiological conditions, such as stress or immune responses, with cortisol acting as a major regulator of the immune system [27,28]. ACTH produced by the activation of the HPA axis will secrete both cortisol and β-endorphin [29]. Cortisol, lysozyme, and β-endorphin are distinctive biomarkers with interconnected roles in neuroendocrine systems and highlight the intricate internal processes of the stress response.

This study aimed to combine behavioral and physiological investigations to gain a more holistic assessment of African green monkey behavior and welfare. We hypothesized that exposure to additional enrichment interventions would result in a better welfare state for the AGMs, as measured by behavioral observations and hair cortisol concentration. To obtain a more objective interpretation of behaviors, this study applied a principal component analysis (PCA) and multivariate generalized linear mixed models to the behavioral data collected from the study population alongside the physiological biomarkers of hair cortisol, beta-endorphin, and lysozyme. This analysis allows for the identification of specific behaviors that have significant associations with physiological stress responses.

## 2. Materials and Methods

A more detailed account of this methodology can be found in the author’s published dissertation [36]. In brief, this study was performed according to good clinical practice standards at the Behavioural Science Foundation (BSF) and Ross University School of Veterinary Medicine (RUSVM). The BSF houses a colony of African green monkeys and is fully accredited by the Canadian Council of Animal Care (CCAC). The study protocol was approved by the RUSVM Institutional Animal Care and Use Committee (IACUC), which is accredited by the Association for Assessment and Accreditation of Laboratory Animal Care International (AAALAC). The Animal Care and Use Committee for the BSF also reviewed and approved the study. The approved collection protocol number 20.04.09 was received on 4 September 2020.

### 2.1. Study Population

The study population comprised wild-caught animals acquired by the BSF for research purposes. A total of 40 adult intact male AGMs (aged 5 to 13 years) were opportunistically sampled and randomly assigned to one of two research groups through an alternating quasi-randomization assignment. The control group (CG) included 20 AGMs that received standard enrichment as provided by the BSF. This standard enrichment consisted of visual, olfactory, and auditory interactions with other conspecifics, a perch, and varied produce as food enrichment, such as banana, pumpkin, and sweet potato, in line with guidance from the Public Health Service (PHS) Policy and the Office of Laboratory Animal Welfare (OLAW). The extra enrichment group (EG), also consisting of 20 AGMs, received additional enrichment in the form of a privacy shield permanently attached to the enclosure and a foraging board with oats provided daily for one hour. All animals were housed outdoors individually during the study period in durable shaded metal enclosures measuring 3′ × 3′ × 4′. The housing enclosure had a squeeze-back mechanism to facilitate safe restraint. The front panel was constructed with evenly spaced, heavy-gauge stainless-steel bars and a securely fastened guillotine door. The extra enrichment group had a thin external panel that measured 1.5′ × 0.02′ × 4′, which served as a privacy shield. The foraging board was externally mounted on the front of the enclosure and featured a flat rectangular surface coated with a textured material to hold treats.

The BSF animal care team was responsible for providing daily care and monitoring of the animals. In accordance with their standard operating procedures, wild-caught AGMs are individually housed during quarantine, following the guidelines set by the Centers for Disease Control and Prevention (CDC). The intake of animals occurs on a continuous rolling basis, and sample collection for this study occurred over a one-year period. Animals were housed outdoors in sturdy metal enclosures that meet or exceed space requirements established by the CCAC. Both groups were fed a standard diet of commercial chow (Teklad NIB Primate diet chow—Inotiv, New Britain, PA, USA) and fresh produce, with access to water ad libitum. From week zero to week two, animals were submitted to an acclimation period to get used to their new surroundings and the observer. Monkeys in the EG received the privacy shield on their first day and foraging board during week one. The collection of behavioral data began on week three and continued to the end of the study period.

### 2.2. Biological Sample Collection

All biological samples were collected on the same morning during weeks 0, 6, and 12 between 8 am and 9:30 am, with consistent methodology (described below) regarding collection and storage. At each timepoint, animals were sedated with ketamine (7–10 mg/kg/IM) in the gastrocnemius muscle.

#### 2.2.1. Blood Collection

As previously described by Roman et al. [36], blood samples were collected from the femoral vein using an appropriate syringe needle. For β-endorphin analysis, 3 mL of blood was placed into an EDTA tube containing 300 μL of Aprotinin, a trypsin inhibitor. Blood samples were centrifuged at 800× *g* for 10 min. The collected plasma was divided into aliquots and frozen at −80 °C until assayed.

#### 2.2.2. Saliva Collection

As previously described by Roman et al. [36], saliva samples were collected after sedation by placing dental cotton in the cheek pouch for a minimum of 5 min. This dental cotton was then centrifuged at 800× *g* for 15 min. The collected material was divided into aliquots and frozen at −80 °C until assayed.

#### 2.2.3. Hair Collection

Hair samples were collected by shaving a 6 cm × 4.5 cm section of hair from their backs with a razor at the root and frozen at −80 °C until assayed, as previously described by Roman et al. (2024) [36].

### 2.3. Biological Sample Analysis

#### Hair Cortisol, β-Endorphin, and Lysozyme Analysis

All kits were validated internally prior to running study samples, as previously described by Roman et al. [36]. β-endorphin levels were assayed from plasma samples following the manufacturer’s directions (Creative Diagnostics^®^ β-endorphin ELISA Kit, Shirley, NY, USA). Lysozyme levels were assayed from saliva samples following the manufacturer’s directions (Creative Diagnostics^®^ LYZ (Human) ELISA Kit, Shirley, NY, USA).

Hair samples were analyzed by a private laboratory (Stress Bioanalytics, Oswego, NY, USA). Their extraction and assay methodology can be found in previously published literature [37,38,39]. Assays were completed using Arbor Assays^®^ cortisol ELISA kits (Ann Arbor, MI, USA).

### 2.4. Behavioral Observations

Behavioral observations were recorded using continuous focal observations by the same individual [40,41]. With this method, an individual animal is watched for a set period, and its behavior is recorded at preset intervals. The only recorded behavior is what the animal is doing at the exact moment the interval ends [42]. Any behavior occurring within the interval is not recorded. For this study, observations took place five times a week, occurring three days in the morning between 7 am and 10:00 am and two days in the afternoon between 2 pm and 5:00 pm. Observation periods were 5 min a day with notation intervals of 10 s. The behavior was coded in an ethogram (Appendix A) adapted from previously published studies [2,41,43,44]. The ethogram included typical behaviors observed in AGMs. The ethogram was further refined during a two-week period of pilot observations before the beginning of the study by adding behaviors previously unlisted.

### 2.5. Statistical Analysis

All data preparation and statistical analysis were completed using R version 4.3.2 run within RStudio version 2023.06.0 + 421 “Mountain Hydrangea” release [45].

#### 2.5.1. Data Preparation

Using the R package dplyr (version 1.1.4) [46], timepoints, hair cortisol concentrations, lysozyme concentrations, and β-endorphin concentrations were added to all 56,000 lines of the behavior observations. Individual behaviors were one-hot encoded using the R package stats (version 4.3.0) [45]. Tetrachoric correlations were run on the binary data using the R package psych (version 2.3.9) to help inform appropriate statistical approaches [47]. Prior to analysis, the biochemical biomarker outliers were defined as being greater or less than 1.5 times the interquartile range [48] and removed from the datasets. One-hot encoded data were aggregated by timepoint before performing principal component analysis (PCA) with the R package caret (version 6.0-94) [49] using the function prcomp with the scale and center arguments set to true. Eigenvalues and cumulative variance percentages were calculated using the R package factoextra (version 1.0.7) [50]. Linear mixed models using R package lme4 (version 1.1-35) [51] and lmerTest (version 3.1-3) [52] were used to assess if there were differences between groups (regarding body weight, behavior, and stress biomarkers) and over time.

#### 2.5.2. Assessing Effectiveness of Enrichment by Hair Cortisol Concentration

Linear mixed models were constructed using R package lme4 (version 1.1-35) [50] and lmerTest (version 3.1-3) [52] to assess the effect of enrichment on hair cortisol while controlling for the weight of the individual monkey (fixed effect) and monkey (random effect). A Gaussian distribution was employed, and the models were tested for assumptions of linearity by plotting the residuals versus plasma cortisol, homogeneity of variance by performing an ANOVA on the calculated squared residuals, independence of residuals by performing the Durbin Watson Test using the R package car (version 3.1-2) [53], and normal distribution of residuals visually through a Q-Q plot and shapiro.test from the R package stats (version 4.4.0) [45]. Post hoc paired comparisons were completed using the R package emmeans (version 1.8.9) [54] to test for significant differences between timepoints.

#### 2.5.3. Assessing Effects of Enrichment and Behavior on Stress Biomarkers

Markov chain Monte Carlo (MCMC) multivariate mixed models in a Bayesian framework were created using the MCMCglmm package (version 2.35) [55] to assess if any behaviors could predict fluctuations in the biochemical parameters while controlling for month, timepoint, and group (enrichment or control). We used an inverse gamma uninformative prior based on the previously published literature [56,57]. For all models, we ran Gaussian distributions with 1,300,000 iterations and a burn-in of 300,000 and sampled the chain every 100–500 iterations, resulting in 2000–10,000 samples of the chain. Plots of the MCMC samples were inspected to ensure convergence of the chains. The biochemical variable lysozyme was square-root-transformed due to the violation of statistical assumptions on the normality of the residuals, while hair cortisol was log10-transformed to ensure Gaussian distribution. The final selected models controlled for month (except for hair cortisol) and monkey and retained only principal components which helped explain the biochemical variable of interest and had the lowest DIC value. The full statistical analysis code and example data are available in the GitHub repository AGMbehavior_biochem [58] (https://github.com/MicroBatVet/AGMbehavior_biochem/tree/main (accessed on 20 February 2025)).

## 3. Results

### 3.1. Descriptive Statistics

Forty male monkeys were enrolled in the study on a rolling basis. There were no significant differences in weights between groups at week 0 (*p* = 0.83), week 6 (*p* = 0.67), and week 12 (*p* = 0.65) (Table 1). There were significant differences between the intake weights and weights at week 6 (*p* <0.01) and week 12 (*p* < 0.01).

### 3.2. Hair Cortisol Concentrations

**Figure 1:**
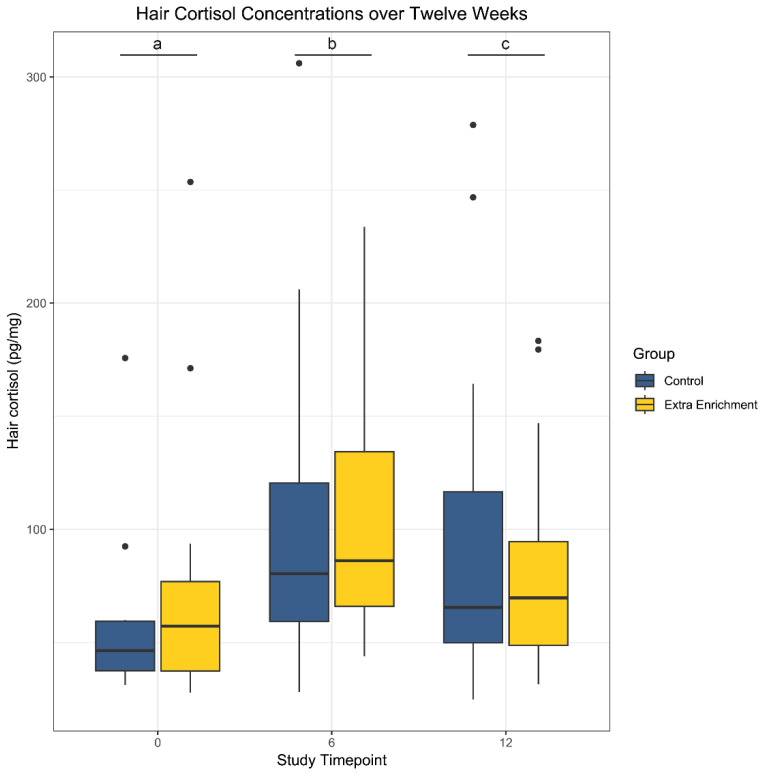
Hair cortisol levels (pg/mL) for the study population. Sample collection occurred at 3 timepoints: week 0, week 6, and week 12. Significance was found when controlling for individual and weight. There are no significant differences between groups but each timepoint was significantly different from each other, denoted by a–c.

Hair cortisol concentrations by timepoint and group are shared in Figure 1. There were 119 observations, and after outlier removal, there were 110 hair cortisol observations. There were no significant differences between the groups. The linear models reveal that the timepoints were significant events, and on post hoc pair-wise comparison, all timepoints were significantly different from each other with *p*-values of less than 0.01.

### 3.3. Principal Component Analysis of Behaviors

**Figure 2:**
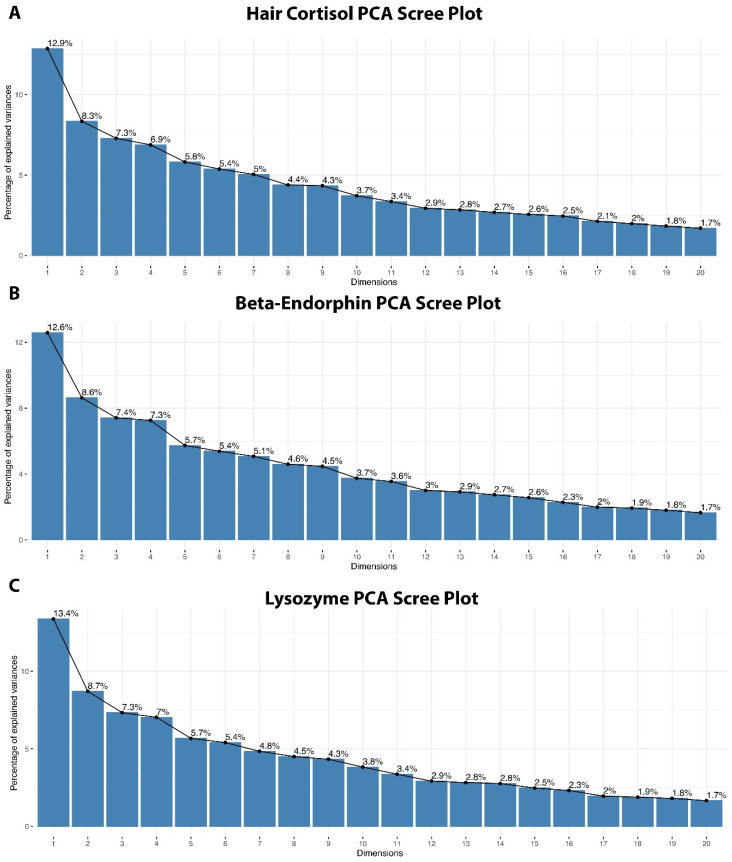
Scree plots showing the first 20 dimensions or principal components for (**A**) hair cortisol, (**B**) β-endorphin, and (**C**) lysozyme biomarker concentrations.

The first 16 PCs explain 80 percent of the variance for hair cortisol (Figure 2A), β-endorphin (Figure 2B), and lysozyme (Figure 2C) concentrations. Appendix B contains the eigenvalues, the percentage of explained variance for each principal component, and the cumulative variance explained for all principal components.

### 3.4. Behavioral Associations with Stress Biomarkers

The following tables present the results of the multivariate mixed models examining the interactions between stress parameters and behavioral loadings (hair cortisol, β-endorphin, and lysozyme in Table 2, Table 3 and Table 4). The tables report the posterior mean, associated 95% confidence intervals, effective sample sizes, and *p*-values. There were no significant differences in behavior between the two groups. For the complete principal component (PC) loadings for all principal components, all contributing behaviors, and observed behavior with sample sizes, refer to the Supplementary Materials File S1. Table 5a–c presents the four top and bottom behaviors that most contributed to each significant principal component of the stress biomarkers. Large loadings (defined by ±0.3) are in bold.

## 4. Discussion

The successful management of stress in captive AGMs is a critical welfare concern due to the physiological and behavioral impacts prolonged stress can have on their well-being and research validity [1,2,3,4]. Ethically, it is the responsibility of the biomedical community to strive for continued refinement of their well-being. Enrichment is a requirement for all NHP populations to improve animal welfare [1,2,3,4]; however, in this study, the chosen enrichment interventions did not produce significant differences between groups. To evaluate their welfare holistically, physiological parameters were evaluated alongside behavioral observations. The hair cortisol analysis provides a retrospective measure of the feral population’s stress levels in situ. The lowest levels were observed upon entry, and the highest levels were observed after 6 weeks, followed by a decrease by 12 weeks with no difference between the groups. Although the enrichment interventions did not have an observable impact on their hair cortisol concentrations, the findings suggest the population was acclimating to captivity. It is important to recognize the distinction between the impact of acute and chronic stress on behaviors. Acute stress can elicit adaptive coping strategies, such as increased vigilance or displacement behaviors, whereas chronic stress can lead to maladaptive behaviors, like stereotypies that indicate poor welfare. Some behaviors may reflect short-term coping mechanisms, but their prolonged expression may suggest chronic stress and would require further attention. It would be beneficial if future research continued to explore the nuances of how acute and chronic stress responses influence behavior.

Effective veterinary management of biomedical NHPs and AGMs requires analysis of both quantitative and qualitative variables [1,2,3,4]. Qualitative variables, like behavior, can be challenging to analyze due to the subjective nature and variability across contexts and species [41]. Generally, management plans aspire to encourage individuals in captivity to express species-specific behaviors like their wild counterparts, with the assumption that similarity equals better welfare [59]. This interpretation is complicated as behaviors are influenced by a multitude of variables, such as the time frame of observation, environmental stimuli, and individuality [40]. The relationship between physiological biomarkers and behaviors is complex, as behaviors can influence physiological processes, while changes in physiological measures can, in turn, drive behavioral changes. NHPs are especially complex compared to other studied species because of their ecological and behavioral flexibility and high psychological and emotional needs [15], all of which can introduce subjectivity to the interpretation of their behavior. This study takes a contextual approach to behavioral interpretation by focusing on behaviors with loading factors (defined by ±0.3) that show significant contributions to fluctuations in physiological biomarkers. We selected the absolute value of 0.3, as many textbooks recommend 0.3 or 0.4 as a minimum loading cut-off value [60,61,62]; however, this is an arbitrary cut-off value. There is no universal agreement among statisticians and researchers on the minimum absolute value for loadings when interpreting principal component loadings [60]. Similarly, there is no uniform way to determine the number of components that should be retained from PCA. Budaev [60] reviews some of the commonly accepted techniques to determine the optimal number of PCs, such as keeping factors with eigenvalues greater than 1.

PCA is a type of machine learning analysis, which is primarily used to condense a large number of variables while still retaining the majority of the variable information used to reduce dimensions [63]. We opted to use PCA as it allowed us to address correlation among all the variables within the dataset [63], as regression analysis assumes the data do not exhibit multicollinearity. The low loading values combined with the low eigenvalues and low percent variance for many of the PCs suggest the behaviors are highly interrelated. Therefore, we opted to retain all PCs and incorporated them into the constructed models, with only the best model retained. This framework allows us to identify specific behaviors as part of a broader analysis of stress-associated patterns that emphasize the complex interplay between behavior and stress physiology.

Some of the observed behaviors are likely driven by immediate environmental conditions like reaching for neighbors, barking, cowering, and fleeing. These behaviors could indicate heightened awareness or engagement with their environment and cohorts. Despite being singly housed, the opportunity to interact socially with their neighbors had a significant impact on β-endorphin levels, suggesting a potential regulatory effect on the stress response and highlighting the importance of social interaction in NHPs. Reaching for neighbors is an affiliative behavior whereas barking, cowering, and fleeing can be defined as agonistic behaviors key to the establishment of social hierarchies. Dominance in hierarchical group settings is often established through aggressive displays, which can activate the stress response, either positively or negatively [64].

Visual and auditory signals, like wiggling ears, further illustrate the influence of external stimuli. Wiggling ears involves a subtle and rhythmic movement of the ears [65]. Visual and auditory signals are important behaviors for NHPs and their ability to communicate socially [65]. Darwin expressed that many NHPs incorporate ear movements into their facial expressions, and these expressions are thoroughly documented in macaques and chimpanzees as behavioral expressions [65]. While there is no specific literature on ear movements for AGMs, the use of this behavior in stressful contexts may serve as a meaningful indicator of a communication tool or indicator of arousal.

Other behaviors in this study may function as coping mechanisms that reflect adaptive strategies to manage arousal or environmental stimuli. Scratching, rolling, inspecting genitals, and self-play may be associated with arousal and serve as displacement behaviors that could help regulate acute stress. Similarly, drinking, while seemingly a straightforward action, can reflect routine hydration, pathological symptoms of diseases like diabetes, or possibly another example of displacement behavior. Conversely, inactivity as a behavior could reflect disengagement, energy conservation, or a neutral state. Enrichment use was unexpectedly associated with higher levels of hair cortisol despite the assumption that enrichment use is generally associated with positive welfare outcomes. This could suggest that the enrichment use also reflected heightened arousal and animals that interacted with the foraging board were utilizing it as a potential coping mechanism.

Another unexpected behavior association was the strong association of pacing with lowering levels of beta-endorphin. Pacing is commonly considered a stereotypic behavior, though emerging research questions this historical classification. Pacing might not necessarily be indicative of poor animal welfare in all cases [8,15,66]. In fact, some evidence suggests that behavior like pacing can be a coping strategy to reduce the negative impact of stress; for example, pacing in captive tufted capuchins was not associated with higher levels of fecal corticoid levels, unlike other stereotypic behaviors like head-twirling [67]. Additionally, studies are beginning to document differences between pacing and agitated locomotion, which could be confused as the same behavior if untrained to the difference. Agitated locomotion is described as rapid, tense, stiff movements, which could be difficult or impossible to differentiate in conditions of single-housing [66]. These findings suggest that pacing is a complex behavior that necessitates deeper understanding. Future research on this topic should aim to understand AGM locomotion styles and their interpretation as coping mechanisms, normal behaviors, or abnormal behaviors, as it relates to the analysis of animal welfare.

Several limitations should be considered when interpreting the findings of this study. The study design was dependent on the standard operating procedures of the research facility and opportunistically enrolled animals as they entered the facility. This led to the rolling admission of a solely male population of feral AGMs with unknown environmental exposures and histories. This introduced potential variability in seasonality and environmental factors that could influence the stress response and animal behavior. The all-male study population introduced another limitation as previous studies have documented behavioral and physiological differences between sexes and age groups within the same species. The male bias restricts the ability to generalize these findings across a broader population. The lack of temperament testing is a limitation of this study as temperament testing can aid in the identification of different personality types thereby facilitating the identification of behavioral trends tied to individual characteristics. Lastly, a human observer opposed to an automated camera system could be an additional stressor, though the individual was trained to be discrete and avoid direct eye contact, and the animals were given time to acclimate to the new environment and observations.

## 5. Conclusions

Enrichment for captive populations is assumed to be beneficial and important, though we cannot assume the effectiveness of specific strategies across species. In this population of AGMs, access to a foraging board and a privacy shield did not significantly alter their welfare state. While this manuscript offers some insights as to why certain behaviors are linked to biochemical values, more study is needed to fully comprehend these complex connections. The objective association of specific behaviors with fluctuations in physiological parameters can provide a more nuanced understanding of behavior. Affiliative and agonistic behaviors could reflect immediate social and environmental conditions that emphasize the critical role of social interactions in stress regulation. Other behaviors may be reflective of high arousal, displacement behaviors, or coping mechanisms.

The promotion of computational ethology stands as a valuable tool offering quantifiable methods to understand and study behavior. The analysis presented demonstrates the effectiveness of combining PCA with a Bayesian mixed model analysis for behavioral assessments of AGMs. Our statistical approach resolves the multicollinearity of all behavioral observations first observed, allowing for a robust analysis of behaviors compared to changes in physiological parameters to better assist in refining animal husbandry and enrichment practices. While it is well-studied that captive animals have a range of behaviors to demonstrate responses to stress, it is less understood how or which behaviors are the result of increasing stress and which may be coping mechanisms. By improving our understanding of how specific behaviors correlate to an animal’s physiology, veterinary management strategies can more effectively assess the effectiveness of enrichment interventions and mitigate stress.

## Figures and Tables

**Table 1:** Animal weights with standard deviations during the study period in kilograms. Weights were significantly lower at week 6 (denoted by *) and week 12 (denoted by ^+^) compared to the intake weight.

Hair Cortisol	Week 0 (kg)	Week 6 (kg)	Week 12 (kg)
CG	5.6 ± 1.1	5.3 ± 0.7 *	5.3 ± 0.7 ^+^
EG	5.7 ± 0.8	5.2 ± 0.7 *	5.2 ± 0.8 ^+^

**Table 2:** The results from the regression analysis examining the interactions between hair cortisol concentrations and behavioral loadings.* Denotes a significant *p*-value equal to or less than 0.05.

Hair Cortisol	Post. Mean	Lower 95% Confidence Interval	Upper 95% Confidence Interval	Eff. Samp	pMCMC
(Intercept)	1.677882	1.549845	1.795571	10,000	<1 × 10^−4^ *
PC4	−0.027003	−0.057058	0.004783	10,000	0.0898
PC7	−0.037074	−0.073222	−0.002166	10,000	0.0404 *

**Table 3:** The results from the regression analysis examining the interactions between β-endorphin concentrations and behavioral loadings.* Denotes a significant *p*-value equal to or less than 0.05.

β-Endorphin	Post. Mean	Lower 95% Confidence Interval	Upper 95% Confidence Interval	Eff. Samp	pMCMC
(Intercept)	2069.874	1636.65	2467.752	2000	<5 × 10^−4^ *
PC4	60.3249	−0.6442	123.799	2000	0.052
PC6	70.1278	−0.4766	147.6484	2000	0.058
PC11	−104.818	−201.881	−2.7427	2000	0.035 *
PC13	−122.691	−232.863	−18.7865	2606	0.025 *
PC26	−174.213	−363.973	22.1145	2000	0.078
PC31	322.7265	68.517	581.9805	2122	0.02 *

**Table 4:** The results from the regression analysis examining the interactions between lysozyme concentrations and behavioral loadings.* Denotes a significant *p*-value equal to or less than 0.05.

Lysozyme	Post. Mean	Lower 95% Confidence Interval	Upper 95% Confidence Interval	Eff. Samp	pMCMC
(Intercept)	1.169086	0.970994	1.380056	2000	<5 × 10^−4^ *
PC11	0.058574	−0.00726	0.128076	2000	0.091
PC15	0.083078	0.013917	0.17204	1744	0.041 *

**Table 5:** (**a**–**c**) presents the four top and bottom behaviors that most contributed to each significant principal component of the stress biomarkers. Large loadings (defined by ±0.3) are in bold.

**(a)**					
**Hair Cortisol**					
**PC7**					
Post. Mean				−0.037074	
**Inspect Genitals**				**0.3445**	
Groom				0.2506	
Flee				0.2439	
Manual Exploration				0.2348	
Oral Exploration				−0.2113	
Vocalize: Chatter				−0.2119	
Eat Feces				−0.2999	
Enrichment Use				**−0.3175**	
**(b)**					
**β-Endorphin**					
**PC13**		**PC31**		**PC 11**	
Post. Mean	−122.691	Post. Mean	322.7265	Post. Mean	−104.818
**Vocalize: Bark**	**0.4112**	**Drink**	**0.4322**	**Self-Play**	**0.3823**
Stare Ahead	0.2516	**Scratch**	**0.4263**	**Scratch**	**0.3530**
Scratch	0.2420	Pounce	0.2155	**Wiggle Ears**	**0.3250**
Drink	0.2249	Pick	0.2138	**Cower**	**0.3076**
Wiggle Ears	−0.1689	Flee	−0.1968	Stare at Neighbor	−0.2033
Stare at Observer	−0.2144	Inspect Genitals	−0.2171	Inspect Genitals	−0.1950
Roll	−0.2422	Defecation	−0.2586	Urination	−0.2719
**Reach for Neighbor**	**−0.5376**	**Pace**	**−0.3407**	**Vocalize: Bark**	**−0.3739**
**(c)**					
**Lysozyme**					
**PC15**					
Post. Mean		0.083078			
**Inactivity**		**0.3911**			
**Vocalize: Bark**		**0.3707**			
**Self-Play**		**0.3056**			
**Roll**		**0.3052**			
Stare Ahead		−0.1615			
Gape		−0.1796			
Cower		−0.2174			
**Flee**		**−0.3265**			

## Data Availability

The raw data supporting the conclusion of this article will be made available by the authors upon reasonable request. Example data as well as the full R code for the data analysis are available at https://zenodo.org/records/12007831 (accessed on 20 February 2025) [58].

## Supplementary Materials

The following supporting information can be downloaded at: https://www.mdpi.com/article/10.3390/vetsci12030209/s1, File S1: Behavioral Loadings.

## Appendix A. Ethogram

**Behavior**	**Description**
Pace	Repeated locomotion, walking back and forth
Flip	Jumping and spinning forward/backward in midair
Drink	-
Eat	-
Sleep	-
Urination	-
Defecation	-
Vocalize—Chatter	Repetitive series of calls
Vocalize—Bark	Aggressive alarm call
Vocalize—Lip smack	Rapid, rhythmic movement of the lips with soft, popping sound
Reach for Neighbor	Extending past enclosure toward adjacent cohort
Stare at Observer	Extended eye contact with observer
Stare Ahead	Alert and watching but not at a specific object and with no movement
Look Around	Vigilantly examining surroundings with moving eyes/head rotation
Inspect Genitals	Looking closely at their own genital region
Groom	Cleaning/maintaining the body using the hands or mouth
Pick	Using fingers to investigate a spot
Scratch	Using hands or feet to rub/scrape skin
Enrichment Use	Interaction with the foraging board
Gape	Sustained wide-open mouth displaying teeth
Oral Exploration	Using mouth and tongue to manipulate/roam enclosure
Manual Exploration	Using hands to manipulate/roam enclosure
Fidget	Restless, repetitive movements (i.e., adjusting posture, tapping, shifting position)
Inactivity	Still, stationary, eyes downcast
Wiggle Ears	Rapid, small movement of the ears
Self-Play	Masturbation
Erect Penis	Displaying genitals in an aroused state
Cower	Submissive posture with lowered body, limbs close to the body, and/or hunched back
Pounce	Sudden quick movement where animal lunges forward
Grab	Attempting to seize an object
Yawn	Mouth opens widely with deep inhalation, followed by closing mouth
Head Twirl	Repetitive motion of the head sometimes referred to as head dance
Roll	Twisting body on bottom of the enclosure to perform a 360 degree rotation
Stretch	Extension of a limb for short period of time
Stare at Neighbor	Eye contact with adjacent cohort
Flee	Rapid, panicked movement away from observer
Eat Feces	Coprophagy
Sneeze	Sudden expulsion of air from the mouth and nostrils
Puff-Up Display	Expansion of the body, hair standing on end to appear larger

## Appendix B. Eigenvalues, Percentage of Explained Variance for Each Principal Component, and the Cumulative Variance Explained for All Principal Components

**Hair Cortisol**			
**eigenvalue**	**variance percent**	**cumulative variance**	**percent**
Dim. 1	4.373180 × 10^0^	1.286230 × 10¹	12.86230
Dim. 2	2.836367 × 10^0^	8.342257 × 10^0^	21.20455
Dim. 3	2.477107 × 10^0^	7.285607 × 10^0^	28.49016
Dim. 4	2.338956 × 10^0^	6.879282 × 10^0^	35.36944
Dim. 5	1.977292 × 10^0^	5.815565 × 10^0^	41.18501
Dim. 6	1.828581 × 10^0^	5.378179 × 10^0^	46.56319
Dim. 7	1.714059 × 10^0^	5.041349 × 10^0^	51.60453
Dim. 8	1.490692 × 10^0^	4.384389 × 10^0^	55.98892
Dim. 9	1.475160 × 10^0^	4.338707 × 10^0^	60.32763
Dim. 10	1.262716 × 10^0^	3.713870 × 10^0^	64.04150
Dim. 11	1.140611 × 10^0^	3.354737 × 10^0^	67.39624
Dim. 12	9.985140 × 10^−1^	2.936806 × 10^0^	70.33304
Dim. 13	9.664956 × 10^−1^	2.842634 × 10^0^	73.17568
Dim. 14	9.126706 × 10^−1^	2.684325 × 10^0^	75.86000
Dim. 15	8.695998 × 10^−1^	2.557647 × 10^0^	78.41765
Dim. 16	8.360252 × 10^−1^	2.458898 × 10^0^	80.87655
Dim. 17	7.224518 × 10^−1^	2.124858 × 10^0^	83.00141
Dim. 18	6.737503 × 10^−1^	1.981618 × 10^0^	84.98302
Dim. 19	6.218685 × 10^−1^	1.829025 × 10^0^	86.81205
Dim. 20	5.772656 × 10^−1^	1.697840 × 10^0^	88.50989
Dim. 21	5.635752 × 10^−1^	1.657574 × 10^0^	90.16746
Dim. 22	5.368461 × 10^−1^	1.578959 × 10^0^	91.74642
Dim. 23	4.140269 × 10^−1^	1.217726 × 10^0^	92.96415
Dim. 24	3.785051 × 10^−1^	1.113250 × 10^0^	94.07740
Dim. 25	3.477195 × 10^−1^	1.022704 × 10^0^	95.10010
Dim. 26	3.229641 × 10^−1^	9.498943 × 10^−1^	96.05000
Dim. 27	2.878527 × 10^−1^	8.466256 × 10^−1^	96.89662
Dim. 28	2.520954 × 10^−1^	7.414571 × 10^−1^	97.63808
Dim. 29	2.141251 × 10^−1^	6.297798 × 10^−1^	98.26786
Dim. 30	1.870804 × 10^−1^	5.502364 × 10^−1^	98.81810
Dim. 31	1.788896 × 10^−1^	5.261459 × 10^−1^	99.34424
Dim. 32	1.264199 × 10^−1^	3.718233 × 10^−1^	99.71606
Dim. 33	9.653793 × 10^−2^	2.839351 × 10^−1^	100.00000
Dim. 34	1.421451 × 10^−31^	4.180737 × 10^−31^	100.00000
**β-endorphin**			
**eigenvalue**	**variance percent**	**cumulative variance**	**percent**
Dim. 1	4.279748 × 10^0^	1.258749 × 10¹	12.58749
Dim. 2	2.934928 × 10^0^	8.632141 × 10^0^	21.21963
Dim. 3	2.517657 × 10^0^	7.404874 × 10^0^	28.62451
Dim. 4	2.466634 × 10^0^	7.254807 × 10^0^	35.87932
Dim. 5	1.947927 × 10^0^	5.729197 × 10^0^	41.60851
Dim. 6	1.828326 × 10^0^	5.377431 × 10^0^	46.98594
Dim. 7	1.722367 × 10^0^	5.065785 × 10^0^	52.05173
Dim. 8	1.564152 × 10^0^	4.600446 × 10^0^	56.65217
Dim. 9	1.520544 × 10^0^	4.472189 × 10^0^	61.12436
Dim. 10	1.273914 × 10^0^	3.746807 × 10^0^	64.87117
Dim. 11	1.207003 × 10^0^	3.550009 × 10^0^	68.42118
Dim. 12	1.020432 × 10^0^	3.001272 × 10^0^	71.42245
Dim. 13	9.925551 × 10^−1^	2.919280 × 10^0^	74.34173
Dim. 14	9.313611 × 10^−1^	2.739297 × 10^0^	77.08103
Dim. 15	8.719866 × 10^−1^	2.564666 × 10^0^	79.64569
Dim. 16	7.749164 × 10^−1^	2.279166 × 10^0^	81.92486
Dim. 17	6.764264 × 10^−1^	1.989489 × 10^0^	83.91435
Dim. 18	6.573791 × 10^−1^	1.933468 × 10^0^	85.84782
Dim. 19	6.113699 × 10^−1^	1.798147 × 10^0^	87.64596
Dim. 20	5.613037 × 10^−1^	1.650893 × 10^0^	89.29686
Dim. 21	5.176059 × 10^−1^	1.522370 × 10^0^	90.81923
Dim. 22	4.616193 × 10^−1^	1.357704 × 10^0^	92.17693
Dim. 23	4.087021 × 10^−1^	1.202065 × 10^0^	93.37900
Dim. 24	3.891853 × 10^−1^	1.144663 × 10^0^	94.52366
Dim. 25	3.355415 × 10^−1^	9.868868 × 10^−1^	95.51055
Dim. 26	2.920089 × 10^−1^	8.588496 × 10^−1^	96.36940
Dim. 27	2.606196 × 10^−1^	7.665282 × 10^−1^	97.13592
Dim. 28	2.453875 × 10^−1^	7.217278 × 10^−1^	97.85765
Dim. 29	2.010271 × 10^−1^	5.912563 × 10^−1^	98.44891
Dim. 30	1.799797 × 10^−1^	5.293520 × 10^−1^	98.97826
Dim. 31	1.637277 × 10^−1^	4.815522 × 10^−1^	99.45981
Dim. 32	1.157071 × 10^−1^	3.403150 × 10^−1^	99.80013
Dim. 33	6.795690 × 10^−2^	1.998732 × 10^−1^	100.00000
Dim. 34	2.383994 × 10^−31^	7.011746 × 10^−31^	100.00000
**Lysozyme**			
**eigenvalue**	**variance percent**	**cumulative variance**	**percent**
Dim. 1	4.54613774	13.3709934	13.37099
Dim. 2	2.95897179	8.7028582	22.07385
Dim. 3	2.49274206	7.3315943	29.40545
Dim. 4	2.39108524	7.0326036	36.43805
Dim. 5	1.92960822	5.6753183	42.11337
Dim. 6	1.83818528	5.4064273	47.51980
Dim. 7	1.64612758	4.8415517	52.36135
Dim. 8	1.53204624	4.5060183	56.86737
Dim. 9	1.47361532	4.3341627	61.20153
Dim. 10	1.30240830	3.8306127	65.03214
Dim. 11	1.14546109	3.3690032	68.40114
Dim. 12	0.99389777	2.9232287	71.32437
Dim. 13	0.96212678	2.8297847	74.15416
Dim. 14	0.94113320	2.7680388	76.92220
Dim. 15	0.84308581	2.4796641	79.40186
Dim. 16	0.79038943	2.3246748	81.72653
Dim. 17	0.66477963	1.9552342	83.68177
Dim. 18	0.64366601	1.8931353	85.57490
Dim. 19	0.61563671	1.8106962	87.38560
Dim. 20	0.56695915	1.6675269	89.05313
Dim. 21	0.50938159	1.4981811	90.55131
Dim. 22	0.45461576	1.3371052	91.88841
Dim. 23	0.39410664	1.1591372	93.04755
Dim. 24	0.37648575	1.1073110	94.15486
Dim. 25	0.33654471	0.9898374	95.14470
Dim. 26	0.30481138	0.8965041	96.04120
Dim. 27	0.27165632	0.7989892	96.84019
Dim. 28	0.23842921	0.7012624	97.54146
Dim. 29	0.22271441	0.6550424	98.19650
Dim. 30	0.18107016	0.5325593	98.72906
Dim. 31	0.15716945	0.4622631	99.19132
Dim. 32	0.11893874	0.3498198	99.54114
Dim. 33	0.08444815	0.2483769	99.78952
Dim. 34	0.07156437	0.2104835	100.00000
